# Traditional milk transformation schemes in Côte d’Ivoire and their impact on the prevalence of *Streptococcus bovis* complex bacteria in dairy products

**DOI:** 10.1371/journal.pone.0233132

**Published:** 2020-05-15

**Authors:** Aimé R. Sanhoun, Sylvain G. Traoré, Kossia D. T. Gboko, Jérôme Kirioua, Fabienne Kurt, Nize Otaru, Patriz Iten, Dasel W. M. Kaindi, Bernd Kreikemeyer, Pierre Renault, Daouda Dao, Jan Hattendorf, Leo Meile, Marina Koussemon, Christoph Jans, Bassirou Bonfoh

**Affiliations:** 1 UFR des Sciences et Technologies des Aliments (STA), Université Nangui Abrogoua, Abidjan, Côte d’Ivoire; 2 Centre Suisse de Recherches Scientifiques en Côte d’Ivoire (CSRS), Abidjan, Côte d’Ivoire; 3 Département Biochimie-Génétique, Université Péléforo Gon Coulibaly, Korhogo, Côte d’Ivoire; 4 École Inter-États des Sciences et Médecine Vétérinaires de Dakar, Dakar, Sénégal; 5 Department of Health Sciences and Technology, ETH Zürich, Zürich, Switzerland; 6 Department of Food Science, Nutrition and Technology, University of Nairobi, Nairobi, Kenya; 7 Institute of Medical Microbiology, Virology, and Hygiene, Rostock University Medical Centre Rostock, Rostock, Germany; 8 Institute Nationale de la Recherche Agronomique, UMR 1319 MICALIS, Jouy-en-Josas, France; 9 UFR Sciences Économiques et Gestion (SEG), Université Félix Houphouët Boigny, Abidjan, Côte d’Ivoire; 10 Department of Epidemiology and Public Health, Swiss Tropical and Public Health Institute, Basel, Switzerland; 11 University of Basel, Basel, Switzerland; University of Torino, ITALY

## Abstract

The *Streptococcus bovis*/*Streptococcus equinus* complex (SBSEC) and possibly *Streptococcus infantarius* subsp. *infantarius* (*Sii*) are associated with human and animal diseases. *Sii* predominate in spontaneously fermented milk products with unknown public health effects. *Sii*/SBSEC prevalence data from West Africa in correlation with milk transformation practices are limited. Northern Côte d’Ivoire served as study area due to its importance in milk production and consumption and to link a wider Sudano-Sahelian pastoral zone of cross-border trade. We aimed to describe the cow milk value chain and determine *Sii*/SBSEC prevalence with a cross-sectional study. Dairy production practices were described as non-compliant with basic hygiene standards. The system is influenced by secular sociocultural practices and environmental conditions affecting product properties. Phenotypic and molecular analyses identified SBSEC in 27/43 (62.8%) fermented and 26/67 (38.8%) unfermented milk samples. Stratified by collection stage, fermented milk at producer and vendor levels featured highest SBSEC prevalence of 71.4% and 63.6%, respectively. *Sii* with 62.8% and 38.8% as well as *Streptococcus gallolyticus* subsp. *macedonicus* with 7.0% and 7.5% were the predominant SBSEC species identified among fermented and unfermented milk samples, respectively. The population structure of *Sii*/SBSEC isolates seems to reflect evolving novel dairy-adapted, non-adapted and potentially pathogenic lineages. Northern Côte d’Ivoire was confirmed as area with high *Sii* presence in dairy products. The observed production practices and the high diversity of *Sii*/SBSEC supports in-depth investigations on *Sii* ecology niche, product safety and related technology in the dairy value chain potentially affecting large population groups across sub-Saharan Africa.

## Introduction

Cow milk plays an important role in pastoralists and urban consumers’ diets across the African continent [1]. For pastoralists, milk and livestock products contribute up to 85% and 63% to their diets and household incomes, respectively [2]. However, homemade processing renders milk particularly perishable and conducive to the spread of pathogenic microorganisms and their toxic metabolites [3–5]. Milk is traditionally fermented to protect it from spoilage, to inhibit outgrowth of pathogenic microorganisms, to prolong shelf life, and to yield popular and regionally-specific fermented dairy products (FDP) in many African countries [1, 6].

During the past decade, *Streptococcus infantarius* subsp. *infantarius* (*Sii*) and *Streptococcus gallolyticus* subsp. *macedonicus* (*Sgm*), both members of the *Streptococcus bovis/Streptococcus equinus* complex (SBSEC), were identified as the predominant lactic acid bacteria (LAB) at 10^8^ CFU mL^-1^ in traditional African FDP, particularly in East African products such as ‘gariss’ in Sudan (camel milk) or ‘suusac’ in Kenya and Somalia (camel milk) but also in ‘fènè’ in Mali (cow milk), and sour milk in Abidjan (southern Côte d’Ivoire, cow milk) [1, 7–10]. SBSEC members and possibly some *Sii* lineages are associated with various human and animal diseases including colorectal cancer, infective endocarditis, bacteraemia and potentially haemorrhoids [11–14]. Unlike human *Sii*, African *Sii* dairy variants present an adapted lactose metabolism similar to that of *Streptococcus thermophilus* based on *lacS* and *lacZ*. Multiple African *Sii* strains are capable of inhibiting certain foodborne pathogens such as *Listeria* spp. However, no explicit virulence factors are described neither for human or food-derived *Sii* [8, 15–17].

Population structure analysis of *Sii* suggests a separation of several dairy lineages from a main presumed pathogenic lineage, but also a shared clade of East African human (EAH) and West African dairy (WAD) strains [14, 18]. Coupled with the predominance in many FDP, these features suggest a pivotal role of *Sii* in African FDP [1]. However, the close relationship of certain West African dairy *Sii* with East African human strains and for some strains also with human blood isolates requires enhanced knowledge of *Sii* prevalence, population structure and potential reservoirs particularly in West Africa [18]. The identification of close contact with livestock and livestock primary products as well as rural habitats as indicators for faecal carriage of *Sii* in humans [19] raises interest in the prevalence of *Sii* in the traditional milk transformation schemes and the production processes associated with them.

Previously, southern Côte d’Ivoire featured a *Sii* prevalence in FDP ranging from 32% to 40% [9]. However, *Sii* prevalence in the north of the country was not assessed, although northern Côte d’Ivoire represents the major cow milk production area with 70% of the national cattle population and the Korhogo region alone is responsible for nearly 50% of its dairy production [20–22]. Northern Côte d’Ivoire with its main city Korhogo is furthermore the first centre of settlement of a ~40,000-people-sized Fulani transhumant population that also inhabit large parts of sub-Saharan West Africa including Mali and Burkina Faso for cross-border trading [23, 24]. Northern Côte d’Ivoire could therefore provide relevant data on cow milk processing and *Sii*/SBSEC prevalence for this large pastoral area of West Africa. Furthermore, the link to production practices is unknown, which is a key aspect regarding the socio-economic impact of milk and FDP for the livelihoods of these populations. Korhogo with approximately 245,000 agro-pastoralists out of the total population of 537,000 [25, 26] provides an ideal study area to assess the prevalence and introduction of *Sii* into the dairy chain. In addition, Korhogo may serve as a representative example for the implementation of a research-to-action study involving dairy stakeholders and policies for sustainable livelihoods for these populations. Therefore, this study aimed to assess the prevalence of *Sii* and other SBSEC bacteria in connection with production and processing practices along the informal cow milk chain in Korhogo.

## Materials and methods

### Growth media and culture conditions of bacteria

Cultivation of lactic acid bacteria and presumptive SBSEC were performed using M17 agar and broth medium (Biolife, Milan, Italy) at 30°C under aerobic conditions and NaCl/peptone diluent (Sigma Aldrich, Buchs, Switzerland) [9]. All media and solutions were prepared using distilled H_2_O and sterilized by autoclaving at 121°C for 15 min.

### Reference strains

*Sii* CCUG43820^T^, *Sii* CJ18, *S*. *thermophilus* DSM 20259, *Enterococcus faecalis* DSM 20478^T^ and *Enterococcus faecium* DSM 20477^T^ were used as reference strains. All strains were obtained from the Culture Collection of the University of Gothenburg (CCUG, Gothenburg, Sweden), Deutsche Stammsammlung von Mikroorganismen und Zellkulturen GmbH (DSMZ, Braunschweig, Germany) and the culture collection of the Laboratory of Food Biotechnology (ETH Zürich, Zürich, Switzerland).

### Sampling of milk products along the supply chain

From January to May 2014, the purpose of the project was shared with all stakeholders prior to a cross-sectional study carried out from May to August 2014. In collaboration with Fulani chiefs and the national laboratory for agricultural developmental support (Laboratoire National d'Appui au Développement Agricole, LANADA), 30 farms representing the starting points of milk supply chains were identified within a radius of 12 km around Korhogo. An additional 30 unfermented milk collectors and 13 artisanal milk product vendors were randomly selected along the milk chains and systematically considered for inclusion. Collectors were not necessary linked to farms or to vendors and vice versa. The inclusion criteria for all the actors were: age above 18 years, agreement to answer the questionnaires and willingness to deliver milk samples.

Milk samples were distinguished into FDP and unfermented milk according to manufacturers’ information and pH measurements. Milk samples were collected into sterile 15-mL Falcon tubes (Bioswisstec, Schaffhausen, Switzerland) at each stage of the supply chain according to the availability of the milk types. Milk samples were stored on ice from field to the laboratory and kept at -20°C prior to analysis [8, 9]. A total of 120 cow milk samples comprised of 48 spontaneously fermented and 72 unfermented milk samples were collected. Milk sample aliquots were taken aseptically to determine the pH values using pH strips (MColorpHast^TM^ pH 4.0/7.0, SigmaAldrich, Buchs, Switzerland) and the temperature using a digital thermometer (DT150, Summit, Inchon, Korea), the environment temperature at the time of sampling and GPS coordinates at each collection site were recorded.

### Survey on milk production practices along the supply chain

Individual questionnaire-based interviews related to milking practices, milk storage, utensils washing, and processing practices were carried out with study participants to collect quantitative data on milk production practices. Observations and group discussions helped to obtain qualitative data. A final restitution workshop involving all stakeholders was organised to define sustainable interventions for the development of dairy value chains in the greater Korhogo region.

### Microbiological analysis of milk samples for SBSEC members

#### Enumeration of presumptive coccoid lactic acid bacteria

Enumeration of coccoid LAB was performed using M17 agar media (Biolife, Milan, Italy) [9] and specific rules for preparation of milk and milk products standard [27]. In short: milk samples were serially diluted and 0.1 mL of dilutions 10^−6^ to 10^−8^ for FDP and 10^−1^ to 10^−3^ for unfermented milk were plated onto M17 agar (Biolife). Plates were incubated aerobically at 30°C for 24 h. Colonies of interest were white or milky, large slimy or pinpoint (0.5 to 1 mm diameter). Plates with 10 to 300 visible colony forming units (CFU) were selected for enumeration.

#### Characterization of presumptive SBSEC bacteria

Isolation and purification of bacteria from M17 agar was performed until isolates were considered as pure by visual streak observation [8]. Gram classification and catalase activity of isolates was determined using 3% KOH and 3% H_2_O_2_, respectively [8].

### Identification of purified bacteria isolates by molecular tools

DNA was extracted from catalase-negative and Gram-positive bacteria single colonies using a simple cell-lysis [28]. General PCR conditions involved 2X PCR master mix (ThermoFisher, Zug, Switzerland), 1 μM final primer concentration and nuclease-free sterile H_2_O to a final volume of 25 μL per reaction. Primers were obtained from Microsynth AG (Balgach, Switzerland).

Molecular identification involved multiple PCR assays. Bacteria DNA was subjected to a multiplex SBSEC-specific PCR assay [29]. Subsequently, isolates were processed using rep-fingerprinting by GTG5 primer [9, 30] for clustering of presumptive duplicate isolates per sample. One isolate per fingerprint type per sample was subjected to *groEL* amplification and sequencing for subspecies identification [14].

Based on rep-fingerprints and sample origin, 24 isolates (23 *Sii* and 1 *Sgm*) were selected to provide maximum diversity to investigate the population structure via a SBSEC-specific multi-locus sequence typing (MLST) scheme and the presence of *lacS/lacZ* genetic adaptations. The MLST scheme was based on the amplification and sequencing of sections of the 10 genes *ddl*, *gki*, *glnA*, *mutS*, *mutS2*, *pheS*, *proS*, *pyrE*, *thrS*, *tpi* [14]. DNA sequence curation and analysis for all applications, MLST analysis and clonal complex definition (7 out of 10 alleles shared with at least one other member) were performed as previously described [14]. DNA-sequence-based phylogenetic trees were constructed by the Maximum likelihood algorithm and 200 bootstrap replications in MEGA 7.0 [31]. MLST sequence and profile data was deposited on www.pubmlst.org.

### Statistical analysis

Data from survey and laboratory analyses were compiled in Microsoft Access 2013 (Microsoft Corporation, Redmond, WA, USA). Descriptive statistics were done using a t-test with 95% confidences intervals (CI) and one factor analysis of variance (ANOVA) allowed to determine the significant influence of different production factors (*p* < 0.05) in SPSS Statistics 20.0 (IBM Corp., Armonk, NY, USA).

### Ethical approval

Ethical approval: All procedures performed in studies involving human participants were in accordance with the ethical standards of the institutional and/or national research committee and with the 1964 Helsinki declaration and its later amendments or comparable ethical standards. The study was approved by the national ethics committee in Côte d'Ivoire (N° 018/MSLS/CNER-dkn) and in Switzerland by the Ethics Committees of ETH Zurich (EK 2013-N-78) and Kantonale Ethik Kommission Zurich (KEK-StV-Nr. 47/14).

Informed consent: Informed consent was obtained from all individual participants included in the study.

## Results

### Milk supply chain organization and processes

#### Milk production system

**Fig 1:**
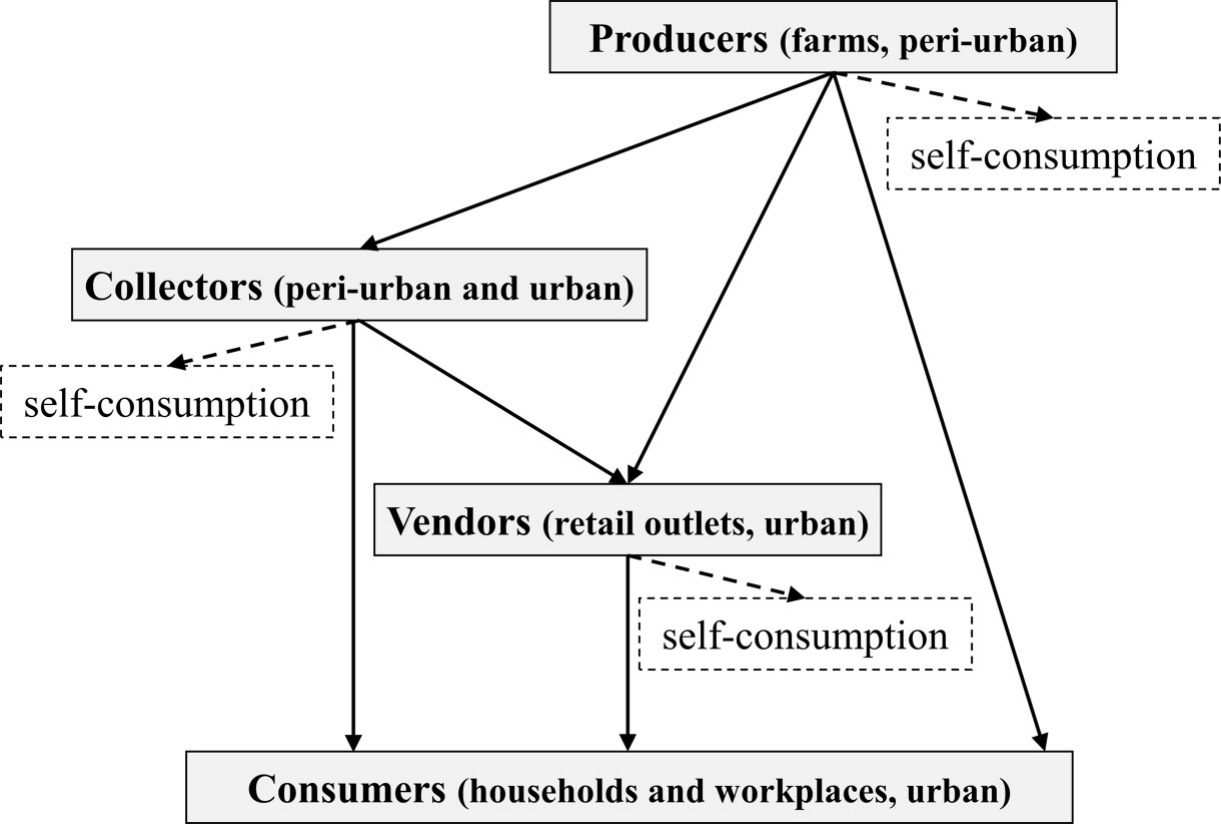
Simplified milk distribution circuit in Korhogo.

From the 30 farms visited, 837 cows were counted. Predominant breeds were 48% crossbreeds [*Bos indicus* x *Bos taurus*]. The daily farm production average was 24.8 L (standard deviation SD 21.1). Out of these, 0.93 L (SD 0.57) (3.6%) were kept for home consumption. The rest was sold through the milk collectors who collect the milk from farm to farm over 40 kilometers or directly in Korhogo town. The produced and collected milk quantities per month were estimated at 744 L and 1959 L for farmers and collectors, respectively. Vendors sold about 1266 L per month. Producers and collectors sold raw cow milk only and vendors sold both fermented and unfermented milk. Metal bowls (16%), calabashes (7%) or plastic containers (77%) were utensils used during hand milking (Fig 1).

#### Socioeconomics of the milk supply chain

The exploratory site visits allowed identification of (i) raw milk producers (n = 30; 73% Fulani; 96.7% men and 3.3% women), (ii) raw milk collectors (n = 30; all Fulani men) and (iii) FDP and unfermented milk vendors (n = 13; 58.3% Fulani; 7.7% men and 92.3% women) as the key groups of the dairy supply chain in Korhogo. Tasks around the milk activities were clearly distributed. The milk production in peri-urban areas and its transportation to town were done by men only while dairy products preparation and selling in retail outlets are mostly women affair indeed. Milk production was a secondary income activity after crop for the producers. In contrast, milk trading was the key activity of collectors who usually gathered in teams of two or three persons.

Two remuneration systems exist between collectors and producers: (i) private collection system with direct purchase with immediate payment (18%) (ii) contractual cooperation system with deferred payment where the value of 20 days goes to the producers and 10 days to the collector (82%). Despite this remuneration system, certain producers directly sold their milk at the price of the market to consumers in town to gain more revenue (Fig 1). The unfermented milk price was 287 XOF/L (SD 11) (€0.44) at the farm, 252 XOF/L (SD 3) (€0.38) at collection stage and 530 XOF/L (SD 24) (€0.81) at the market indeed. The exchange rate was 655.957 XOF for 1€. The monthly revenues were estimated at 83,100 XOF (SD 13,242) (€127) for producers, 88,821 XOF (SD 19,669) (€135) for collectors and 176,000 XOF (SD 22,223) (€268) for vendors.

#### Milk processing techniques

**Fig 2:**
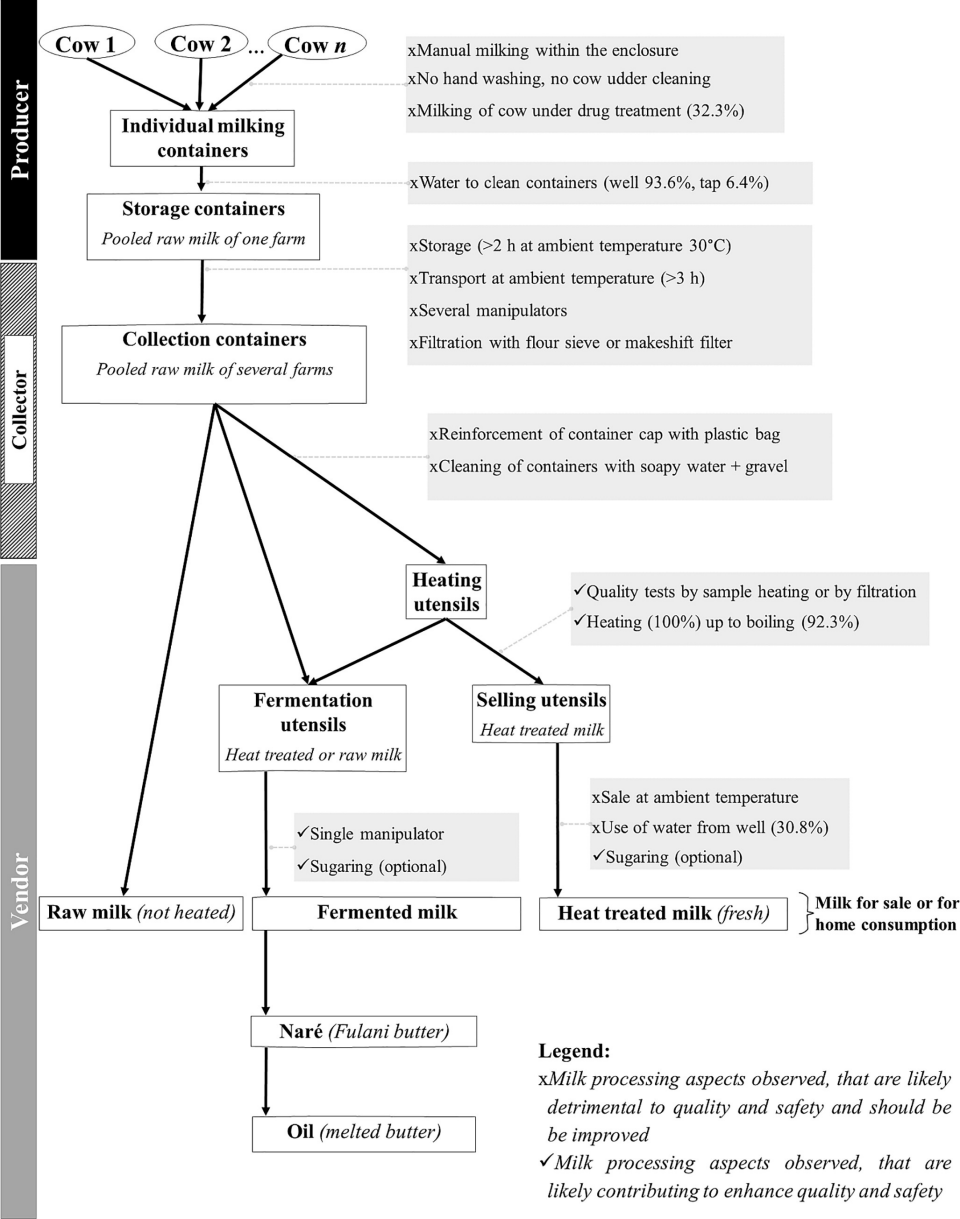
Summary chart of the production and the processing of cow milk in Korhogo along the supply chain as observed during this field study.

Milk processing relied on secular sociocultural practices. This was observed for several key quality and safety aspects along the production chain (Fig 2): Producers and collectors (86.7% Fulani) didn’t filter (51.6%) the milk. Raw milk consumption was common due to ancestral beliefs and particularly common at producer and collector level. They stated ‘*In Fulani tradition*, *if milk is heated*, *cow’s udders dry up and animals die*. *In addition*, *raw milk brings more strength*.’ Furthermore, general aspects of hygiene and good manufacturing practices were lacking: Hand-washing and udder cleaning were not observed, storage (> 2 h) and transportation (> 3 h) were performed at ambient temperature, improper oil or chemical containers were used for milk storage, improper cleaning techniques of containers and 1/3 of producers observed were milking of cows under antibiotic treatment without respecting the withdrawal times.

Despite the traditional raw milk production, heating of milk was observed for some vendors heat-treated or fermented milk products. For heating, the majority (>90%) of vendors heated the milk to boiling temperatures. However, the heat-treated milk was still sold at ambient temperatures (31.5°C) by the majority of vendors. Only approximately 1/3 of vendors sold their milk refrigerated. Vendors kept the milk raw only on specific demand by customers.

For fermented products, all the study participants kept the milk in cleaned individual utensils for 17–19 h at home at room temperature. Only vendors produced fermented milk for sale. Producers and collectors produced fermented milk only for home consumption. The initiation of fermentation at vendor level (vendors n = 9) was based on backslopping (n = 6), the addition of commercial/industrial yogurt from heated milk (n = 1), a combination of both termed “seeding” (n = 2) or spontaneous fermentation. Approximately 1/3 of vendors declared to use spontaneous fermentation practices depending on environmental conditions. During the dry season, the warm room temperature (~29°C) [32] sustained the spontaneous fermentation and delivered FDP in about 17–19 h. In the rainy season, the room temperature was relatively low (under 25°C) [32] and fermentation took much longer, so the milk was initiated via backslopping or through the addition of an industrial yogurt as described above.

The degree of fermentation depended on the desired organoleptic properties of the final product such as flavour, texture and acidity. The fermentation can be continued for 48 to 72 h hours at ambient temperature and after an efficient churning, vendors obtained a Fulani butter called Naré. By heating, they then turned the Naré into oil (melted butter). Both Naré and oil are used for consumption and body ointment (Fig 2).

### Physicochemical parameters and bacterial loads analysis of milk products

At producer level, the pH of unfermented milk was 6.9 (SD 0.1) (Table 1). After average transportation times from farms to vendors of 5h 42min at ~31.1°C, the pH was significantly reduced to 6.7 (SD 0.2) (95% CI) at vendor level in town. For FDP, the lowest pH value of 4.1 (SD 0.2) was recorded at the vendor level, but no difference was observed compared to pH values of 4.6 (SD 0.6) and 4.2 (SD 0.2) of fermented milk from producers and collectors, respectively (Table 1).

**Table 1:** Physicochemical parameters and bacterial counts on M17 agar of milk products collected in Korhogo.

Milk supply chain level	Milk type (number of samples)	Milk pH [mean pH (SD)]	Milk temperature [mean °C (SD)^†^]	Storage temperature [mean °C (SD)^†^]	Cell count [M17, mean Log_10_ CFU mL^-1^ (SD)^†^]
**Peri-urban/Producer**	Unfermented (30)	6.9 (0.1)^a^^§^	32.5 (2.7)^a^	30.0 (2.2)	3.3 (0.9) ^a^
	Fermented (29)	4.6 (0.6)^A^^§^	29.4 (1.2)^A^	30.0 (2.2)	6.9 (2.4) ^A^
**Peri-urban and city/Collector**	Unfermented (30)	6.8 (0.3)^b^^§^	31.8 (2.6)^a^	32.1 (1.9)	5.4 (0.9) ^b^
	Fermented (5)	4.2 (0.2)^A^^§^	31.8 (0.8)^B^	33.4 (2.1)	7.1 (0.7) ^A^
**Markets/Vendor**	Unfermented (12)	6.7 (0.2)^b^^§^	42.7 (11.8)^b^	31.5 (1.9)	3.1 (1.3) ^a^
	Fermented (13)	4.1 (0.2)^A^^§^	30.4 (2.7)^AB^	31.6 (1.9)	6.4 (2.7) ^A^

^†^SD: standard deviation; pH, temperatures and bacterial counts not connected by the same superscript lower-case letter (a, b, c) or capital letter (A, B, C) are significantly different (alpha = 0.05); lower-case letters are for tests for unfermented milk and capital letters for fermented milk within the same column. Storage temperature was not tested for significant differences.

^§^pH: values were determined using pH strips, therefore pH values and statistics on pH differences should be considered as indicative and not absolute values.

The initial bacterial load of 3.3 log_10_ CFU mL^-1^ (SD 0.9) of the raw milk increased significantly (*p* < 0.05) while passing from producer level to collector level (5.4 log_10_ CFU mL^-1^; SD 0.9). Bacterial counts on M17 agar of FDP samples from producers (6.9 log_10_ CFU mL^-1^; SD 2.5), collectors (7.1 log_10_ CFU mL^-1^; SD 0.7) and vendors (6.4 log_10_ CFU mL^-1^; SD 2.7) were little different (Table 1). Out of the 120 milk samples collected, 10 samples (eight from vendors and two from producers) did not provide any presumptive LAB colonies on M17 agar after repetitive plating.

### Phenotypic characterization of presumptive SBSEC isolates

A total of 527 bacteria were collected from M17 agar medium used to analyse 110 milk samples. Out of these isolates, 45.7% and 54.3% originated from fermented and unfermented milk samples, respectively. Out of 527 isolates, 16 isolates were excluded due to catalase-positive examination and two isolates due to fungi growth yielding 509 Gram-positive and catalase-negative isolates.

### Molecular identification of presumptive SBSEC isolates to assess milk sample prevalence

The 509 isolates were processed by rep-fingerprinting to identify 494 representative isolates for processing by the SBSEC-specific PCR assay. Thereby, 178 isolates originating from 58 samples were identified as members of the SBSEC. Among these SBSEC isolates, 105 isolates originated from 29 out of 43 fermented milk samples (67.4%), while 73 isolates originated from 30 out of 67 unfermented milk samples (44.8%) (Table 2).

**Table 2:** Prevalence of SBSEC bacteria and their subspecies in fresh (n = 67) and fermented milk samples (n = 43) and yielding 178 SBSEC isolates out of 286 fresh milk and 241 fermented milk isolates.

		Number of samples				
Species	Sample Type	Producer (n/n_total_)	Collector (n/n_total_)	Vendor (n/n_total_)	total number (n/n_total_, % of total per sample type)^†^	Number of isolates (n/n_total_, % of total per sample type)
***Sii***	Unfermented	13/30	9/29	4/8	26/67 (38.8)	54/286 (18.9)
	Fermented	18/28	2/4	7/11	27/43 (62.8)	85/241 (35.3)
***Sgm***	Unfermented	0/30	5/29	0/8	5/67 (7.5)	8/286 (2.8)
	Fermented	2/28	1/4	0/11	3/43 (7.0)	2/241 (0.8)
**Other SBSEC (species not determined)**	Unfermented	0/30	0/29	0/8	0/67^‡^	11/286 (3.8)
	Fermented	2/28	0/4	0/11	2/43 (4.7)	18/241 (7.5)
**Total SBSEC**	Unfermented	13/30	13/29	4/8	30/67 (44.8)	73/286 (41.0)
	Fermented	20/28	2/4	7/11	29/43 (67.4)	105/241 (59.0)

^†^A total of three fermented and one fresh milk samples harboured *Sii* and *Sgm*. To obtain the number of samples positive for *Sii* and *Sgm*, these samples were counted once for each species. As a consequence, %-values for sample prevalence exceed 100%.

^‡^No fresh milk samples were observed that only harboured SBSEC, instead they were either categorized as *Sii* or *Sgm* samples. Individual SBSEC isolates were obtained from samples with *Sii* or *Sgm* status.

After further representative reduction according to rep-fingerprints and sample origin (data not shown), 47 isolates were identified by partial *groEL* sequencing as *Sii* (n = 42) and *Sgm* (n = 5). Through clustering isolates by rep-fingerprints and association of *Sii* or *Sgm* species to individual clusters, a total of 139 and 10 isolates were assigned subsequently to *Sii* and *Sgm*, respectively. Remaining 29 SBSEC isolates were not further identified to species level. Sequence identities of *groEL* fragments ranged between 99.6–99.8% over 542 bp for *Sii* and 99.8–100% over the same 542 bp to *Sgm*. Two isolates displayed highest sequence identities of 95.0% and 96.4% to *Sgm* and *Sii*, respectively, but were identified as *Sii* via MLST (S1 Fig).

*Sii* were isolated from FDP (27/43) and unfermented (26/67) milk samples yielding a relative prevalence of 62.8% and 38.8%, respectively (Table 2). The highest *Sii* sample prevalence was observed among FDP at producer level where 18 out of 28 samples (64.3%) yielded *Sii* (Table 2). FDP yielded 62.8% (50.0–64.3%) *Sii* sample prevalence in contrast to unfermented milk samples at 38.8% (31.0–50.0%) along the three sample stages (Table 2). Overall, *Sii* represented 26% (n = 139, 85 from fermented milk and 54 from unfermented milk) of all 527 isolates, whereas *Sgm* and SBSEC of unknown subspecies represented only 5.5% and 2.3, respectively (Table 2). The *lacS/lacZ* adaptation was identified in 44 *Sii* isolates out of 90 isolates tested. Of these 44 *Sii*, 26 were isolated from FDP while 18 originated from unfermented milk samples. Out of 18 samples (14 FDP and four unfermented milk samples), 20 isolates were identified as non-SBSEC members harbouring the *lacS/lacZ* adaptation.

### SBSEC population structure by MLST

**Fig 3:**
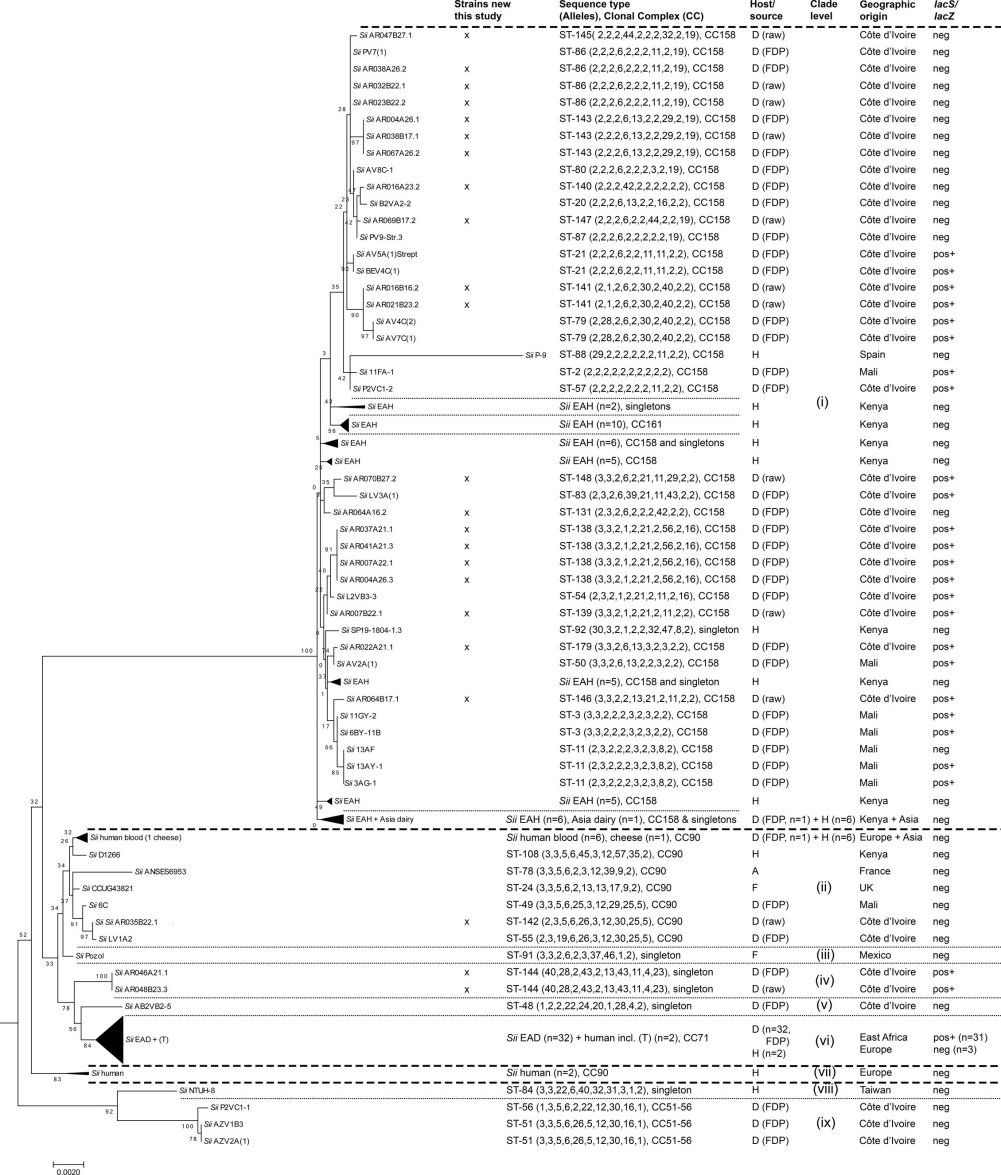
Sequence-based MLST tree of *Sii* isolates of dairy and human origin. The tree was constructed using the concatenated sequences of all 10 MLST loci of all isolates and calculated using the Maximum likelihood algorithm. The tree was extracted from the combined SBSEC tree rooted to *S*. *alactolyticus* DSM 20728^T^ (S1 Fig). Host and sources are indicated for dairy (D), human (H), animal (A) and food (F) and precision for fermented dairy products (FDP) or raw milk (raw). Clade numbers and levels were defined according to tree hierarchy. The percentage of trees in in which the associated taxa clustered together is shown next to the branches calculated from 200 bootstrap replications. The tree is drawn to scale, with branch lengths measured in the number of substitutions per site and indicated by the bar below the graph.

The population structure of 23 *Sii* isolates and one *Sgm* isolate was analysed by SBSEC MLST (Fig 3). Isolate selection was aimed at representing different sample origins and sample types. Out of these 24 isolates, 12 *Sii* isolates harboured the *lacS/lacZ* adaptation, six of which originated from FDP and six from unfermented milk samples. The majority (18 out of 23) *Sii* isolates analysed in this study clustered in clade (i) (Fig 3). Thereby, clade (i) predominantly comprised isolates of CC158. CC158 in total was comprised of 39 WAD, 1 Asian dairy and 40 EAH isolates. Out of these isolates, *lacS/lacZ* genes were only detected in 23 WAD *Sii* but not among the remaining members of clade (i). Other WAD *Sii* isolates harbouring *lacS/lacZ* were only detected in clade (iv) (Fig 3). Novel clades (iv) and (v) thereby represent two lineages potentially separating from the main WAD/EAH clade (i, CC158), the presumed pathogen clade (ii, CC90) and the EAD clade (vi, CC71) (Fig 3). Out of the 23 *Sii* isolates analysed in this study, only *Sii* AR035B22.1 clustered among the presumed pathogen clade (ii, CC90).

The MLST sample set of this study of 23 *Sii* isolates included four *Sii* pairs. Each pair was comprised of an isolate from FDP and one from unfermented milk samples originating from the same study participant households (AR007, AR016, AR038 and AR064). Two pairs (AR007A22.1 & AR007B22.1 and AR038A26.2 & AR038B17.1) featured identical *lacS/lacZ* status within each pair and clustered in closely related branches. In contrast, the other two pairs (AR064A16.2 & AR064B17.1 and AR016A23.2 & AR016B16.2) featured mixed *lacS/lacZ* status, minimizing the possibility for a close relationship between the individual pair members. Furthermore, two *Sii* isolates originated from the same sample but featured the *lacS/lacZ* adaptation only in one isolate (AR004A26.3), which suggests the co-existence of multiple *Sii* lineages within products and households.

Among the *S*. *gallolyticus* branch, the only *Sgm* isolate (AR061A17.2) obtained from WAD products in this study clustered in CC37 and featured the same ST-40 as an isolate previously obtained from FDP in Abidjan (S2 Fig). No *Sgp*, *Sgg* or other SBSEC species representing colonies were obtained in this study.

## Discussion

This study described the milk production system, including the milk supply chain and FDP production in correlation with the prevalence of *Sii*/SBSEC bacteria for sub-Saharan pastoral areas on the example of Korhogo, Northern Côte d’Ivoire.

The milk supply chain in Korhogo is governed by the informal system as in the greater Sudano-Sahelian area [33–35]. The clear separation of the tasks around the milk activities is common in the traditional family farm system predominated by Fulani in this area [36]. Milk trading was the dominant activity for collectors and vendors. This is in contrast to farm owners, who consider milk revenues of the existing remuneration system of as complementary means to remunerate their Fulani herdsmen. This finding is shared with other urban and peri-urban areas such as in and around Bamako (Mali) [35, 37, 38].

Milk production quantities reflected the importance of Korhogo in the dairy sector of Côte d’Ivoire and possible the Sudano-Sahelian area. The high milk amount produced per farmer per month in Korhogo of over 700 L compared to Bamako with 600 L and Abidjan (south Côte d’Ivoire) with 300 L might be related to fodder availability and advanced breeding capabilities. Combined with a larger cow population in northern Côte d’Ivoire, this area is thus considered as the dairy basin, featuring not only high production but also high consumption quantities of milk [20–22, 35, 39].

Production practices in the African setting have been reported to pose risks of microbial contamination of milk [37, 40, 41]. In this study, dairy production practices were assessed to not comply with basic food hygiene standards like the Codex Alimentarius [42]. Instead, they were influenced by secular sociocultural practices around milk [37, 43–45]. Multiple potential sources of contamination were identified: (i) the lack of hygienic practices such as the non-washing of the hands of the milkers and the udders of cows, the milking of cows in the pens, (ii) the permanent contact of the utensils with the ground and the faeces of animals, (iii) the massive presence of insects, (iv) the type of material used for milking, storage and transportation that are difficult to clean such as calabashes or plastic cans, (v) the non-heating of milk at producer and collector levels, (vi) the combined effects of the long storage and transportation at hot ambient temperature until delivery to sellers and customers as well as (vii) the washing system. These factors or the combination thereof seem to have favoured multiplication of *Sii*/SBSEC in the raw milk [39, 44–47]. Particularly, the consumption of raw milk by study participants and the idea that raw milk is “better” for their health as previously reported [37] indicates a health risk [3–5, 48]. The systematic heating of the raw milk at vendor level contributed to a significant reduction of the bacterial load of presumptive LAB in the unfermented milk. However, cell counts of 10^3^ CFU/mL after heat treatment suggests recontamination after heating or improper heating, supporting observations for possible non-compliance with good manufacturing practices as well as the potential re-inoculation of milk with *Sii*/SBSEC via utensils [10, 45]. This would also explain why *Sii* or SBSEC sample prevalence was not largely impacted by the heating step at vendor level.

In FDP, the lack of significant differences in bacterial counts in the supply chain is likely related to the fermentation process, which is usually yielding comparable bacterial counts for other West African FDP [9, 10]. However, randomly distributed FDP samples (n = 10) across the supply chain, where no bacteria were recovered decreased statistical significance of differences. Particularly the absence of LAB such as enterococci, lactococci and streptococci on M17 agar otherwise expected in spontaneous FDP samples with a pH <4.5 [8]. Recurrent reasons for such cell count variations might be explained by the treatment of FDP by (i) a bacteria elimination step such as sufficient heating masked by the addition of potash salts to reduce the coagulation of acid milk during the heating, (ii) the addition of acidic material such as baobab fruit that can result in a low pH, modify the predominating microbiota without the high presence of LAB or (iii) administration of antibiotics into the milk as previously reported [37, 39, 49]. Such administration of antibiotics would be a high public and animal health risk given global spread of antibiotic resistances and the prevalence of multiple zoonotic pathogens in dairy products as well as that of *Sii*/SBSEC [46, 50–52].

In total, almost 2/3 of all FDP and nearly half of all unfermented milk samples were positive for SBSEC strains. To the best of our knowledge, no studies have so far reported the presence of *Sii* strains in unfermented milk [1, 8–10]. However, *Sii* could have been present in the milking containers before the milking as reported for other bacteria and may be linked to cross-contamination from FDP through utensils and handlers, or they may have originated from animals and the environment [10]. This theory is supported by our finding that *Sii* were present in unfermented milk samples collected in the respective containers just after the milking procedure. Detailed analysis of samples from the dairy production environment will be necessary to identify the ecological niche of *Sii*/SBSEC at the livestock-human-milk interface.

The current state of knowledge supported by population structure analysis via SBSEC MLST at the livestock-human-milk interface is not yet conclusive on the origin and niche of *Sii*. While some strains suggest the potential of contamination such as for participant households AR007 and AR038, households AR016 and AR064 suggest the opposite. Clearly, MLST analysis displayed discrimination between the main WAD/EAH and likely commensal *Sii* clade (i, CC158)), presumed pathogen clade (ii, CC90) as well as a newly separated dairy-adapted WAD *Sii* clade (iv, singletons) closer related to that of dairy-adapted EAD *Sii* clade (vi, CC71). This suggests the separation of the WAD *Sii* across these three clades and CCs This supports the previous observations regarding the high strain diversity among West African *Sii* isolates in contrast to *Sii* isolates of East Africa [14]. To which degree this implies health risks for the consumers remains unclear and will require further investigations.

The finding of high overall prevalence of SBSEC strains in spontaneously FDP and unfermented milk samples adds the area of Korhogo in northern Côte d’Ivoire to the places of confirmed *Sii* and SBSEC presence in West Africa in addition to Mali and Abidjan area of Côte d’Ivoire [9, 10]. This high overall prevalence might contribute to livestock and livestock primary product contact and rural habitat as indicators for *Sii* faecal carriage in humans [19]. Given the transhumant populations and cross-border trade in northern Côte d’Ivoire, this supports the postulated hypothesis of an even wider distribution of *Sii* across sub-Saharan Africa [1]. Relating to microbiota data from similar raw and fermented dairy products in Southern Côte d’Ivoire, Mali, Kenya, Sudan and Somalia [7–10], this suggest that a similar role of *Sii* and *Sgm* in the dairy fermentation process could be also be expected for the area of Korhogo. This supports the urge to assess the ecology of these bacteria in the dairy and connected livestock environment in West Africa and elucidate their role in dairy fermentations in comparison to *S*. *thermophilus*.

### Conclusion

This study provided the first dairy production system assessment and *Sii*/SBSEC prevalence determination for Northern Côte d’Ivoire, the main dairy producing area of Côte d’Ivoire. The dairy production system featured limited compliance with good manufacturing practices. SBSEC bacteria, predominantly *Sii*, were found to be present in around 2/3 of FDP and over 1/3 of unfermented milk samples. *Sii* were present as dairy-adapted and non-adapted variants. This adds northern Côte d’Ivoire and likely further areas in West Africa connected via cross-border trading routes to the areas of confirmed *Sii* presence in dairy products. Therefore, *Sii* and SBSEC members likely have an impact on the diet of dairy consumers across multiple countries of sub-Saharan Africa. Furthermore, *Sii* population structure and presence of dairy adaptation marker genes suggest ongoing diversification and evolution into a novel dairy-adapted West African *Sii* lineage. This novel lineage formed in addition to previously described CC158 (clade (i)) of WAD and EAH *Sii* of presumed commensal origin and that of CC90 harbouring mainly human blood isolates (clade (ii)). This high diversity and shared relationships with potential commensal as well as presumed pathogenic clades support in-depth investigations of the ecology and public health risks of *Sii*/SBSEC lineages in the dairy environment and their role in comparison to *S*. *thermophilus*.

## Supporting information

S1 Fig Sequence-based MLST tree of SBSEC of dairy, animal and human origin. The tree was constructed using the concatenated sequences of all 10 MLST loci of all SBSEC isolates and calculated using the Maximum likelihood algorithm. The tree was rooted to *S*. *alactolyticus* DSM 20728^T^. The main species clades are indicated for *S*. *infantarius* subsp. *infantarius*, *S*. *lutetiensis* and the *S*. *gallolyticus* branch with geographic origin of isolates specifically indicated for West Africa (WA) and East Africa (EA). The percentage of trees in which the associated taxa clustered together is shown next to the branches calculated from 200 bootstrap replications. The tree is drawn to scale, with branch lengths measured in the number of substitutions per site and indicated by the bar below the graph.
(TIF)

S2 Fig Sequence-based MLST tree of the *S*. *gallolyticus* branch with isolates of dairy and human origin. The tree was constructed using the concatenated sequences of all 10 MLST loci of all isolates and calculated using the Maximum likelihood algorithm. The tree was extracted from the combined SBSEC tree rooted to *S*. *alactolyticus* DSM 20728^T^ (S1 Fig). Host and sources are indicated for animal (A), dairy (D), human (H), West African Dairy (WAD), East African Dairy (EAD) and fermented dairy products (FDP). Clade numbers and levels were defined according to tree hierarchy. The percentage of trees in in which the associated taxa clustered together is shown next to the branches calculated from 200 bootstrap replications. The tree is drawn to scale, with branch lengths measured in the number of substitutions per site and indicated by the bar below the graph.
(TIF)
