# Supplementary figures and images for: Traditional milk transformation schemes in Côte d’Ivoire and their impact on the prevalence of Streptococcus bovis complex bacteria in dairy products
Source: PLoS One. 2020 May 15;15(5):e0233132. doi: 10.1371/journal.pone.0233132 (PMC7228116; doi:10.1371/journal.pone.0233132)

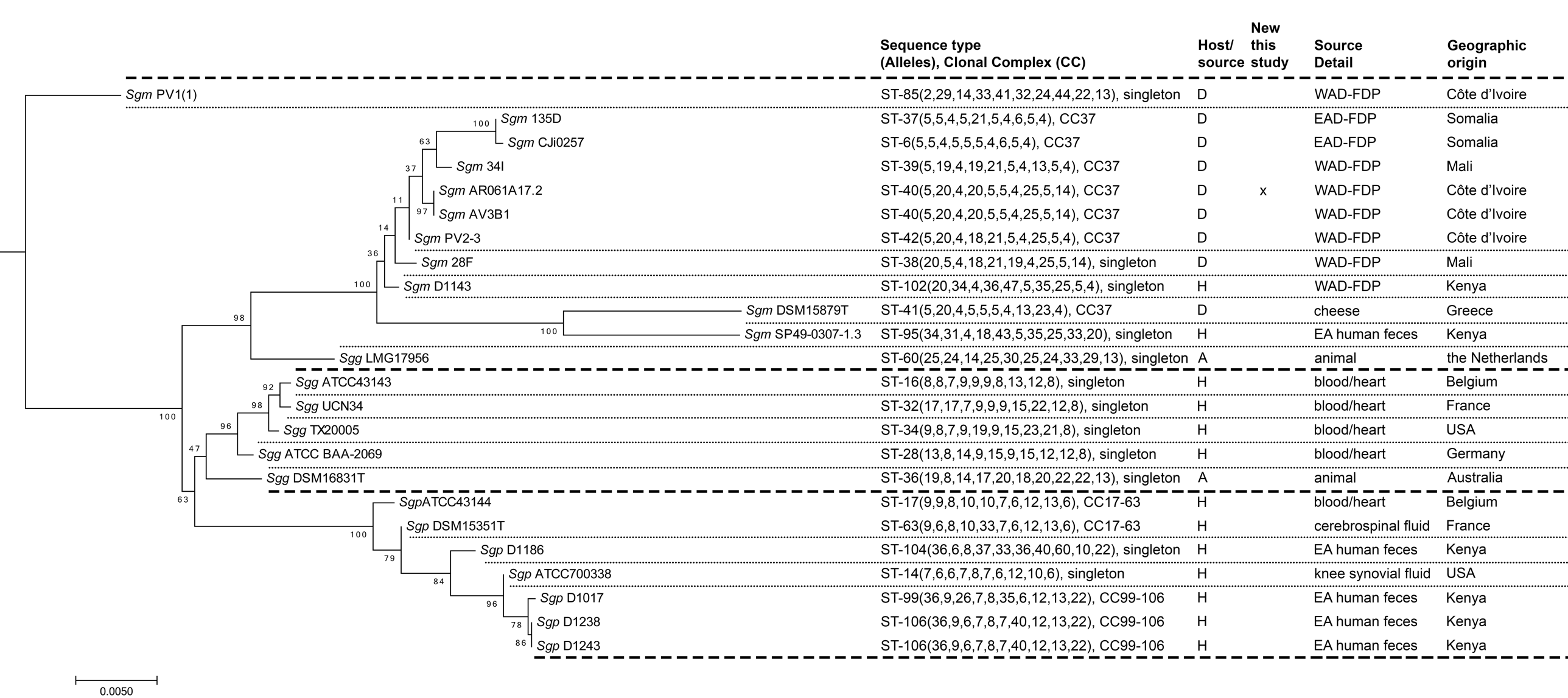

Supplement: S2 Fig — The tree was constructed using the concatenated sequences of all 10 MLST loci of all isolates and calculated using the Maximum likelihood algorithm. The tree was extracted from the combined SBSEC tree rooted to S. alactolyticus DSM 20728T (S1 Fig). Host and sources are indicated for animal (A), dairy (D), human (H), West African Dairy (WAD), East African Dairy (EAD) and fermented dairy products (FDP). Clade numbers and levels were defined according to tree hierarchy. The percentage of trees in in which the associated taxa clustered together is shown next to the branches calculated from 200 bootstrap replications. The tree is drawn to scale, with branch lengths measured in the number of substitutions per site and indicated by the bar below the graph. (TIF) [file pone.0233132.s002.tif]
